# Supplementary material for: Microscopic structure of the polymer-induced liquid precursor for calcium carbonate
Source: Nat Commun. 2018 Jul 3;9:2582. doi: 10.1038/s41467-018-05006-w (PMC6030133; doi:10.1038/s41467-018-05006-w)
Supplement: Supplementary file 1 — Supplementary Information [file 41467_2018_5006_MOESM1_ESM.pdf]

Supplementary Information for

Microscopic structure of the polymer-induced liquid precursor for calcium carbonate

Xu et al.

Supplementary Figures

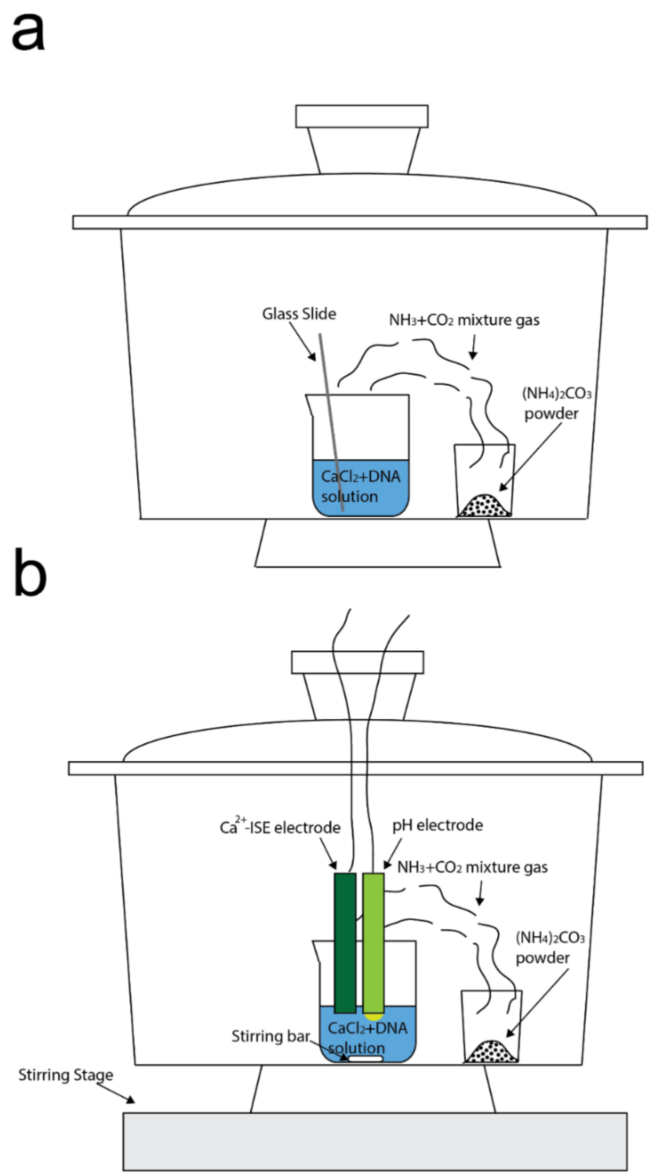

Supplementary Figure 1. Scheme of the experimental setup for $CaCO_3$ mineralization.[1] (a) Reaction without stirring. A beaker containing solution with 10 mM $CaCl_2$ and 2.5 g L^{-1} of ds-DNA, or 1g L^{-1} of pAH, or 25 mg L^{-1} of pAsp was placed in a desiccator. A hydrophilic glass slide was vertically inserted into the solution as the substrate for $CaCO_3$ growth. A vial containing $(NH_4)_2CO_3$ powder was placed near the solution. The CO_2/NH_3 mixture gas released by $(NH_4)_2CO_3$ decomposition was slowly diffused into the solution to induce the $CaCO_3$ formation. (b) Reaction with slow stirring (100 rpm). pH electrode and Ca^{2+}-ion selective electrode (ISE) were directly inserted into the reaction solution for measurements.

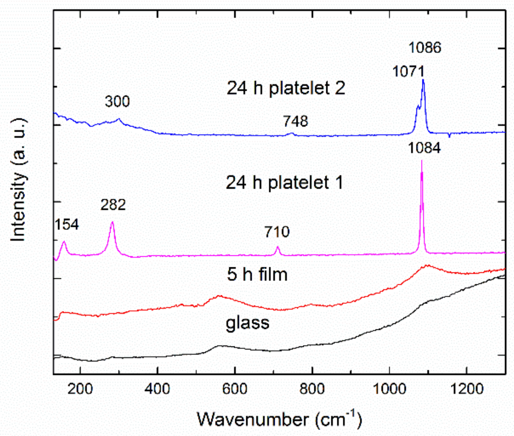

Supplementary Figure 2. Raman spectra taken on the products grown with 25 mg L^{-1} of pAsp. The spectrum of the glass substrate is also shown for comparison. The glass substrate shows 3 humps around 500-600, 800 and 1085 cm^{-1}, respectively. The 3 humps could also be observed for the thin film grown for 5 h, indicating that part of the signal is from the glass substrate. However, the hump at 1085 cm^{-1} increases significantly, suggesting the formation of ACC.[2] The rhombic platelet found at 24 h (platelet 1) showed peaks corresponding to the CO_3^{2-} ν_1 symmetric stretch mode (1087 cm^{-1}), ν_4 symmetric vibration mode (710 cm^{-1}) and external modes (154 and 282 cm^{-1}) of calcite. The round shaped platelet found at 24 h (platelet 2) showed peaks corresponding to the split CO_3^{2-} ν_1 symmetric stretch mode (1071 and 1087 cm^{-1}), ν_4 symmetric vibration mode (748 cm^{-1}) and external mode (300 cm^{-1}) of vaterite.[3, 4]

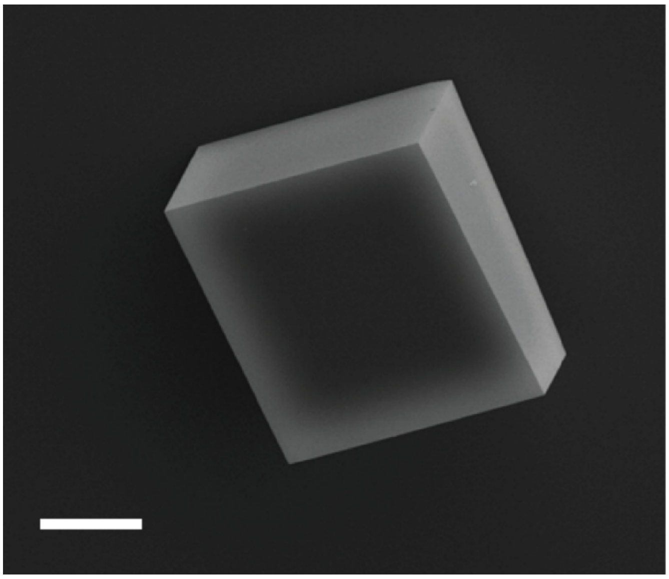

Supplementary Figure 3. SEM image of a calcite crystal grown after 30 mins without additive, which is ~20 µm in size and shows well-facet rhombohedral morphology. Scale bar: 5 µm.

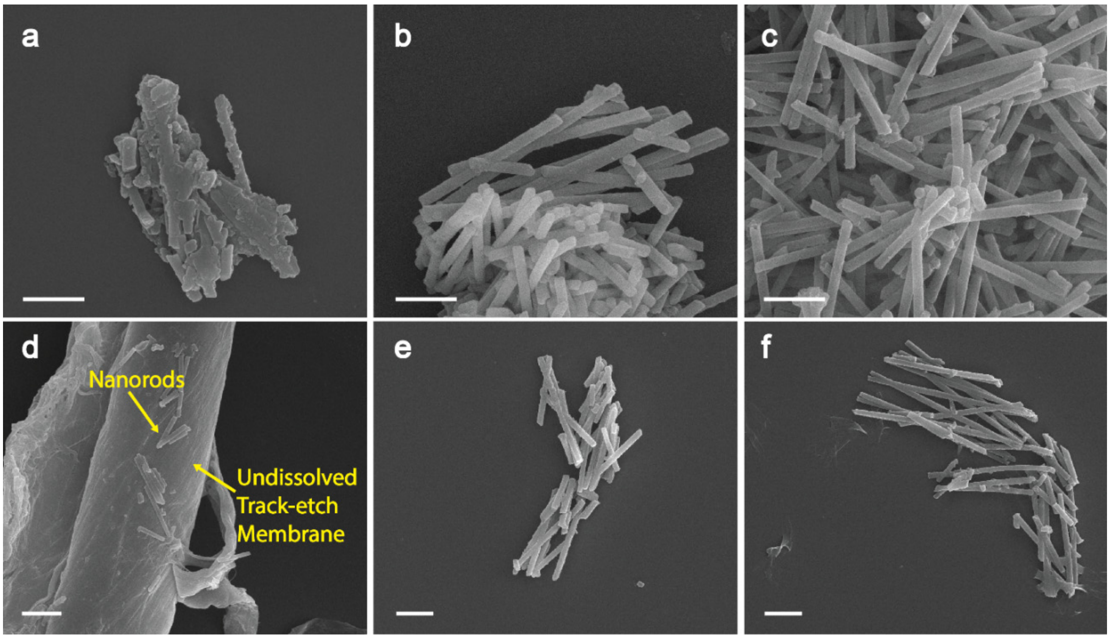

Supplementary Figure 4. SEM images of the $CaCO_3$ nanorods grown in 10 µm thick track-etch polycarbonate membranes, with pore size of 50 nm in (a-c) or 200 nm in (d-f). The samples were grown (a, d) without additive, (b, e) with 2.5 g L^{-1} of ds-DNA, or (c, f) with 25 mg L^{-1} of pAsp. The nanorods and undissolved track-etch membrane are highlighted respectively by yellow arrows in (d). The results show that the presence of ds-DNA or pAsp increases both the amounts and lengths of the $CaCO_3$ nanorods grown within track-etch membranes (see also Supplementary Table 1). Scale bars: (a-c), 1 µm. (d-f), 2 µm.

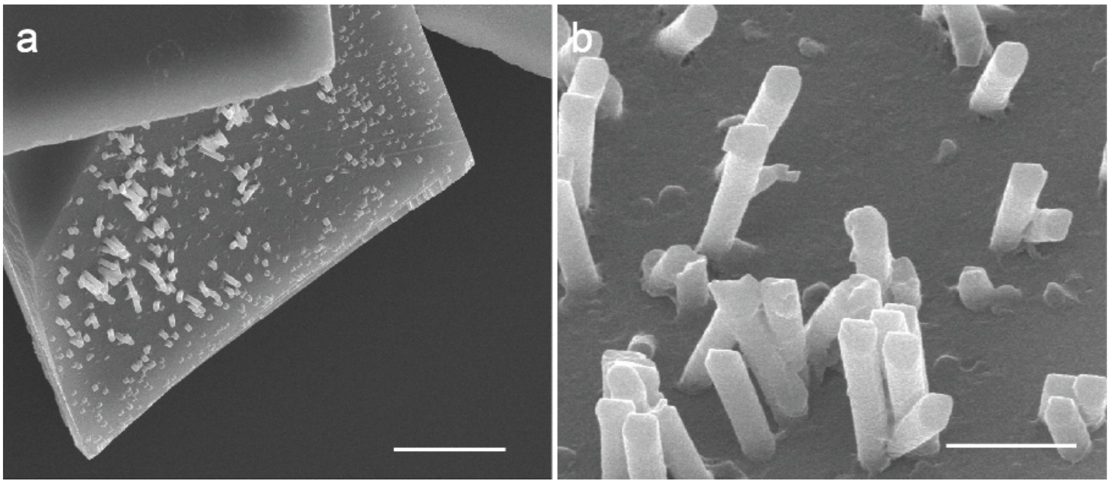

Supplementary Figure 5. (a-b) SEM images of the $CaCO_3$ nanorods developing from the calcite single crystal surface into 200 nm sized nanopores, in the absence of additives. (b) shows that the nanorods are cylinder-shaped and with crystal facets on their tips. Scale bars: (a) 5 µm, (b) 1 µm.

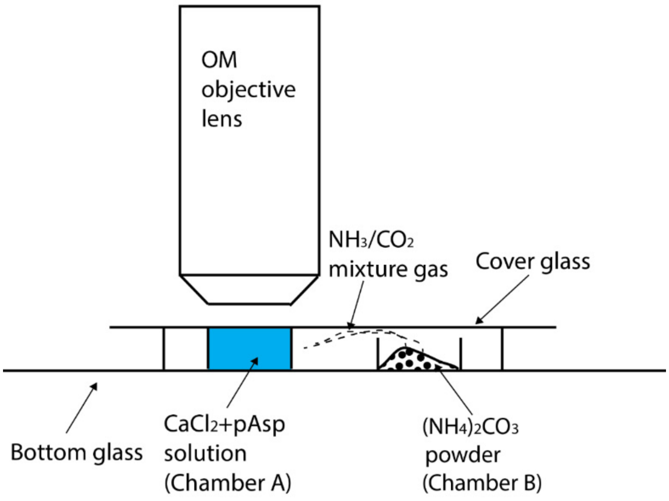

Supplementary Figure 6. Scheme of the in-situ OM observation experiments.

In-situ optical microscopy observations of $CaCO_3$ thin film formation process were performed in a specifically designed growth chamber (Supplementary Fig. 6) similar as what used by Gower et al.[5] In the growth chamber, 0.12 g of $(NH_4)_2CO_3$ was loaded in a lower cell B. The CO_2/NH_3 mixture gas released by $(NH_4)_2CO_3$ decomposition was slowly diffused into a higher cell A containing 39 mL of reaction solution with 10 mM $CaCl_2$ and 25 mg L^{-1} of pAsp. The products were formed on the cover glass and observed using optical microscope.

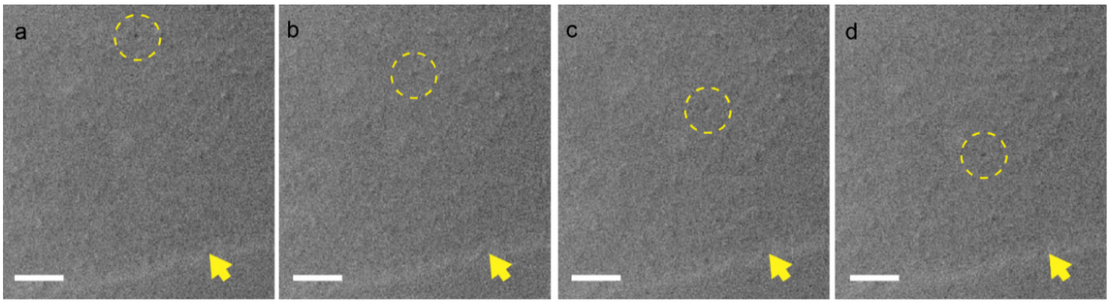

Supplementary Figure 7. DICOM images taken after the reaction started for 182 min, showing a ~2 µm sized particle (highlighted by the yellow circle) flowing on the solution/glass interface during the thin film formation. (a-d) were taken at 0, 18, 42, 75 sec, respectively. The boundary of the thin film is highlighted by yellow arrow. Scale bars: 20 µm.

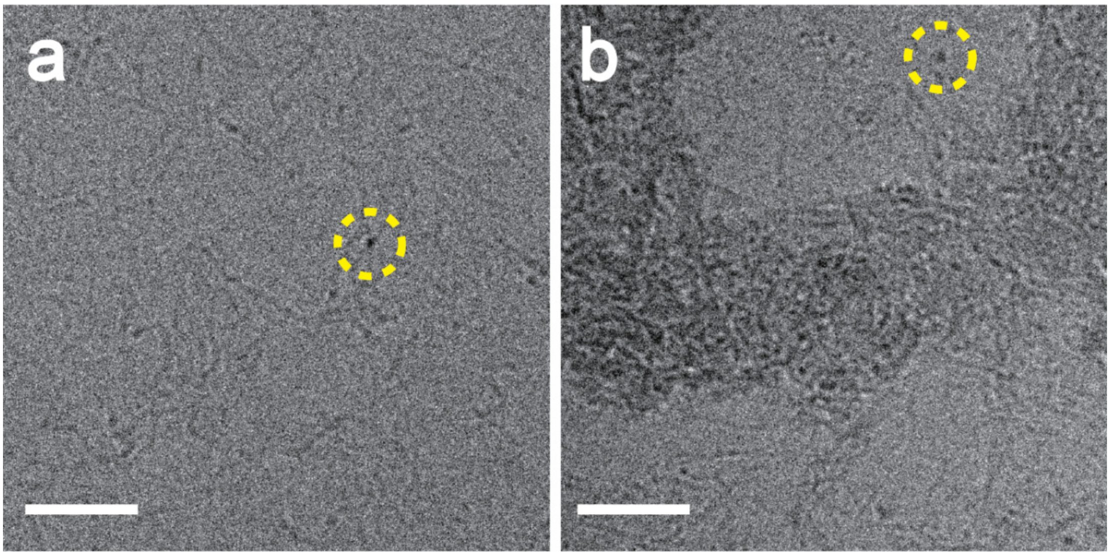

Supplementary Figure 8. (a) CryoTEM image of 2.5 g L^{-1} ds-DNA dispersed in a pH=7.4 phosphate buffer solution. (b) Aggregation of ds-DNA molecules found in the solution after 10 mins of reaction. Black spots corresponding to ds-DNA molecules viewed head-on are highlighted by yellow circles in the images. Scale bars: 50 nm.

In the experiments with 25 mg L^{-1} of pAsp, no liquid-like droplets or any other product was observed by cryoTEM in the solution before the NPs shown in Fig. 2a. In the experiments with 2.5 g L^{-1} of ds-DNA, before the formation of NPs shown in Fig. 2d, the solutions were indistinguishable from the pH=7.4 phosphate buffer solution containing 2.5 g L^{-1} of ds-DNA (Supplementary Fig. 8a). Some aggregates of ds-DNA were occasionally found in the solution (Supplementary Fig. 8b) due to the binding with Ca^{2+} that neutralized the zeta potential (Supplementary Table 5). Although some ~2 nm sized black spots were observed, similar spots also exist in the ds-DNA/buffer solution, and were not visualized in the pAsp experiments. These spots are attributed to ds-DNA molecules viewed head-on.[6]

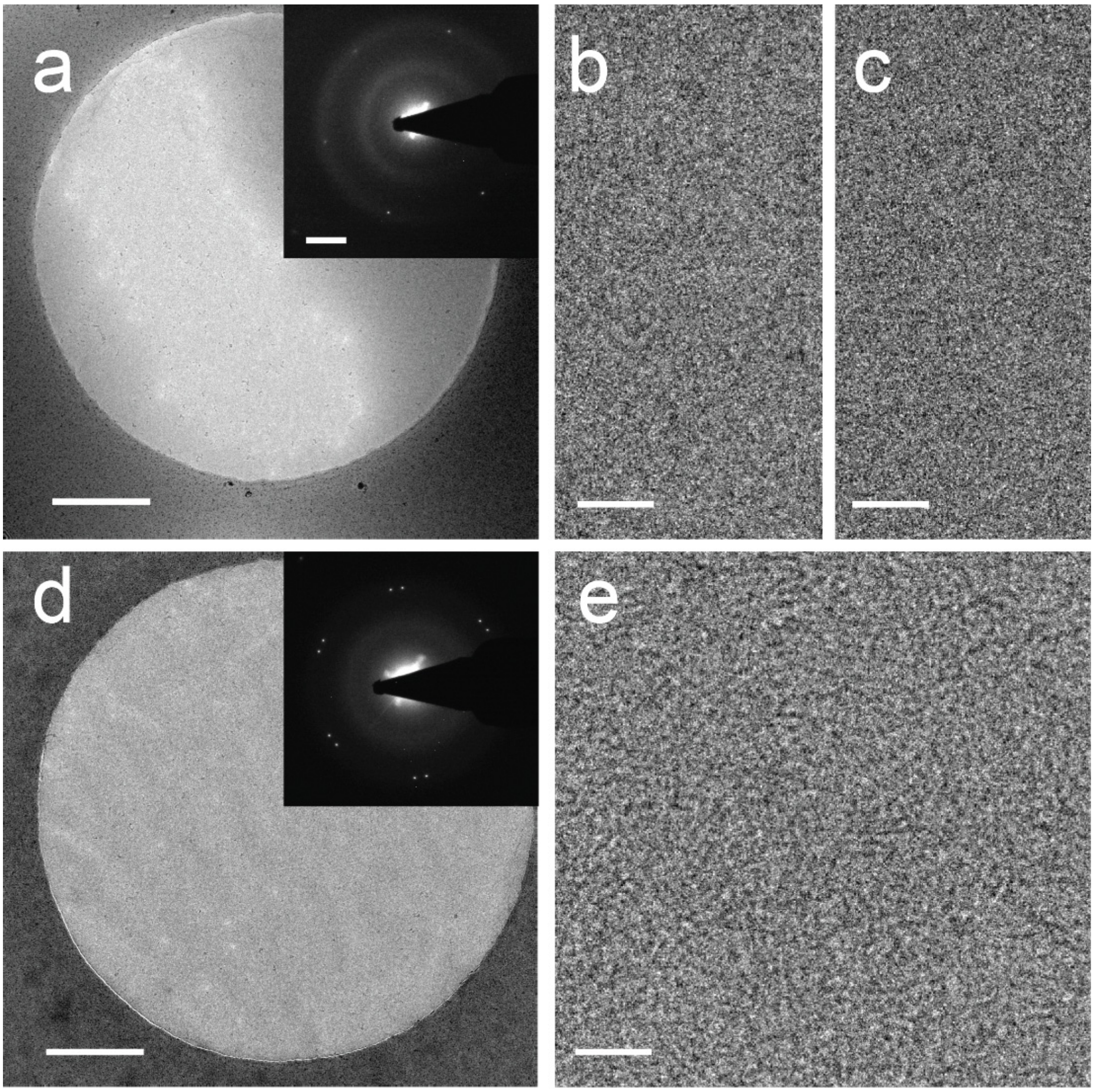

Supplementary Figure 9. CryoTEM images of early stage reaction solutions prepared on GOx supported TEM grids. (a-b) Reaction solution with 25 mg L^{-1} of pAsp after 90 min of experiment. (a) shows the overview of the ice layer. The SAED pattern in the inset shows the diffraction signal of GOx monolayer, or several layers in the same orientation. (b) shows the zoom-in image, which shows only noise-like signal similar to the flat-field (empty) image as shown in (c). (d-e) Reaction solution with 2.5 g L^{-1} of ds-DNA after 15 min of experiment. (d) shows the overview of the ice layer. The SAED pattern in the inset shows the diffraction signal of GOx layers in two slightly misaligned orientations. (e) shows the zoom-in image, which shows densely packed ds-DNA molecules. The higher density of ds-DNA is due to the 60 seconds of waiting time applied during the vitrification, which allows the ds-DNA molecules to condense on the GOx layers. (b) and (e) are both taken with a defocus value of -1.5 µm. Scale bars: (a, d) 500 nm, insets of (a, d) 2 nm^{-1}, (b, c, e) 20 nm.

To further check the existence of earlier products such as individual ACC cluster or liquid droplets, we applied the earlier products (15 min for ds-DNA experiment and 90 min for pAsp experiment) on graphene oxide (GOx) coated TEM grids. This recently developed method allows the formation of much thinner vitrified ice layers (~10 nm),[7] which could greatly improve the contrast of cryoTEM images. Still no product was detected in these experiments as shown by Supplementary Fig. 9. The extremely low concentration of individual clusters or smaller assemblies suggests that they are not stable before assembling on the polymers.

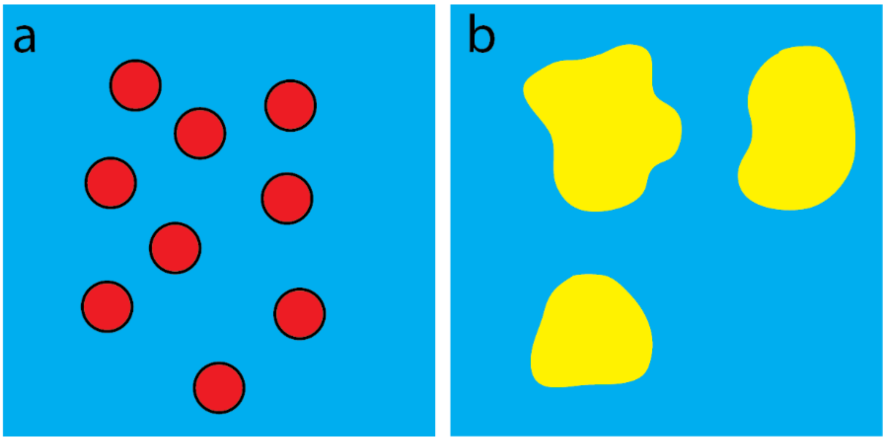

Supplementary Figure 10. Scheme for the morphological difference of liquid droplets dispersed within another liquid. (a) For droplets with small size and/or high liquid-liquid interfacial energy, their shape will be close to spherical in order to minimize the surface area and reduce the interfacial energy. (b) For droplets with large size and/or low liquid-liquid interfacial energy, their shapes will be more easily disrupted and thus are more irregular.

In the ideal case without considering the gravity and small local inhomogeneity, the equilibrium shape of liquid droplets dispersed within another liquid is spherical.[8] This is in order to minimize their surface area and reduce the liquid-liquid interfacial energy. For the droplets with smaller size (higher surface area ratio) and/or high liquid-liquid interfacial energy, their shape will be strongly regulated by the interfacial energy and thus close to spherical (Supplementary Fig. 10a). For droplets with bigger size (lower surface area ratio) and/or lower liquid-liquid interfacial energy, however, the shapes will be more easily disrupted by the blotting and plunging during the vitrification, as well as gravity and local fluctuation of density, temperature or ionic strength, etc. As a result, although still being continuous objects with smooth edges, their shapes become irregular (Supplementary Fig. 10b).

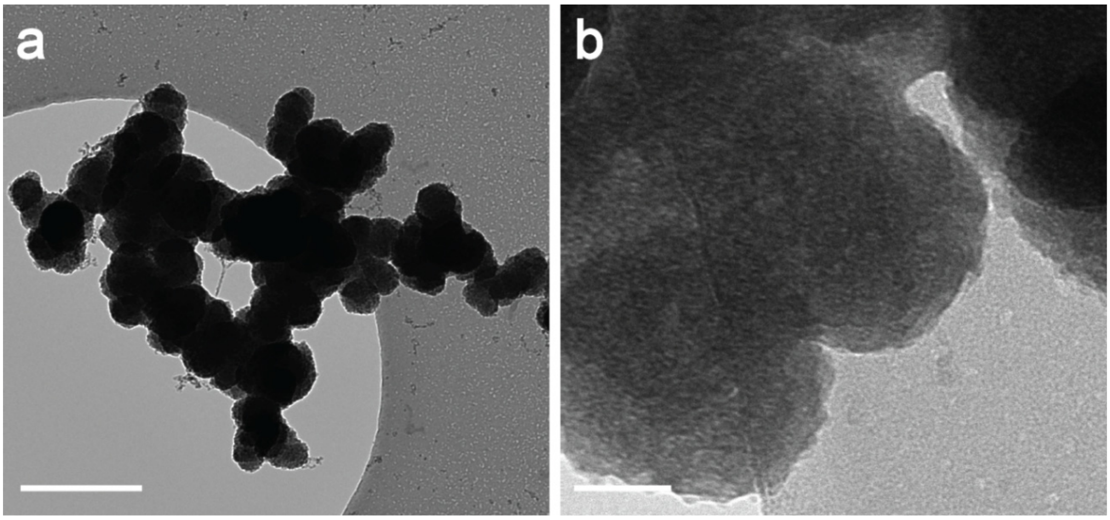

Supplementary Figure 11. TEM images of freeze dried samples of the pAsp-induced NPs (250 min), showing morphology similar to the images taken before freeze drying (Fig. 2c). Scale bars: (a), 500 nm. (b), 50 nm. Similar morphologies were previously observed by dry TEM for the early product of acid polysaccharide induced ACC thin films.[9]

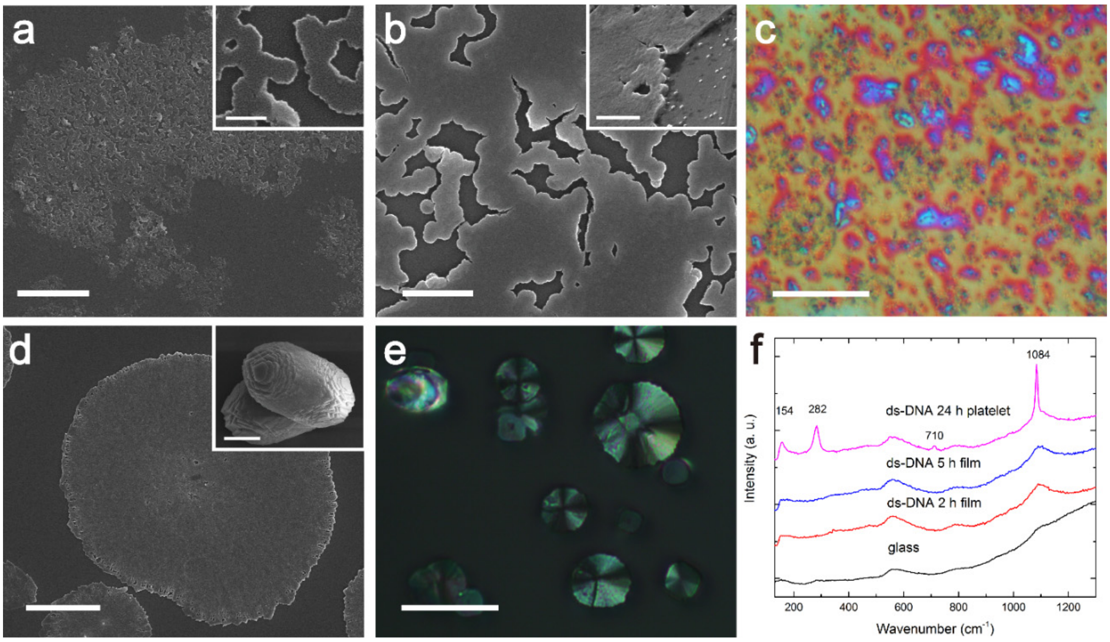

Supplementary Figure 12. The $CaCO_3$ thin film formation process with 2.5 g L^{-1} of ds-DNA and 10 mM $CaCl_2$. (a) SEM image of the thin film formed on glass slide at 2 h. Inset is a zoom-in image showing the film is aggregated by ~80 nm sized nanoparticles, which is consistent with the observation in Fig. 2f. (b) SEM image of $CaCO_3$ thin film grown with 2.5 g L^{-1} of ds-DNA for 5 h, the inset shows the cross section of the thin film after tilting for 45°, revealing a thickness of 400 nm. (c) OM image of the 5 h film using reflecting mode without polarization. The membrane was colorful due to its thickness which fell in the range of visible light wavelength (390-780 nm). (d) SEM image of a platelet formed at 24 h. Inset shows a 3D product. (e) Cross polarized optical microscope (cPOM) image of the platelets. The platelets were bright and shows Maltese-Cross pattern, indicating they were spherulitic crystals. (f) Raman spectra taken on the products grown with 2.5 g L^{-1} of ds-DNA, with the spectrum of the glass substrate shown for comparison. The glass substrate shows 3 humps around 500-600, 800 and 1085 cm^{-1}, respectively. The thin film of 2 and 5 h show similar 3 humps, indicating that the signals are partly from the glass substrate. The increase of signal at 1085 cm^{-1}, however, suggests the formation of ACC.[2] The platelet found at 24 h showed peaks corresponding to the CO_3^{2-} ν_1 symmetric stretch mode (1087 cm^{-1}), ν_4 symmetric vibration mode (710 cm^{-1}) and external modes (154 and 282 cm^{-1}) of calcite.[3, 4] Scale bars: (a) and (d), 5 µm. (b), 2 µm. (c) and (e), 20 µm. Inset of (a), 200 nm.

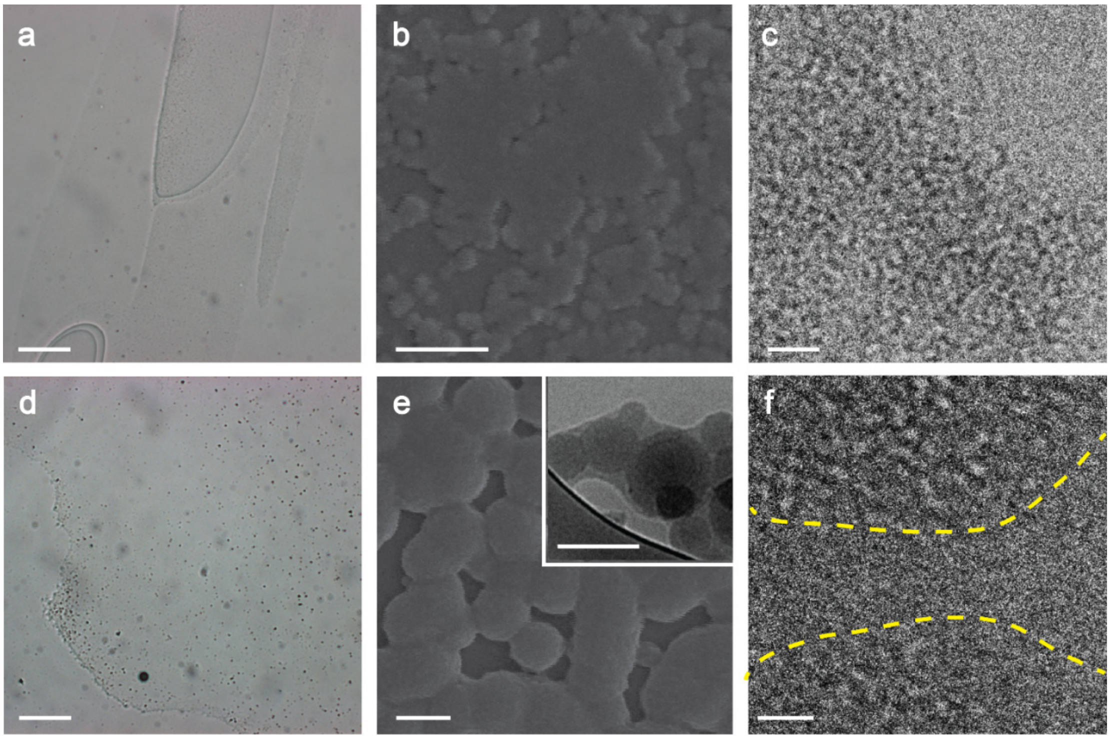

Supplementary Figure 13. PILP processes induced by pAA or pAH. (a-c) PILP induced by 25 mg L^{-1} of pAA with 10 mM of Ca^{2+}. (a) OM image showing the ACC thin film formed on the glass slide at 3 h. (b) SEM image of the thin film, showing ~50 nm sized NPs on the surface of the thin film. (c) CryoTEM image of the product collected at 3 h, showing the ~2 nm sized nanoparticulate texture of the NPs. (d-f) PILP induced by 25 mg L^{-1} of pAH with 10 mM Ca^{2+}. (d) OM image showing the ACC thin film formed on the glass slide at 3 h. (e) SEM image of the thin film, showing ~200 nm sized NPs on the surface of the thin film. Inset is a cryoTEM image of the surface reaction solution at 3 h, showing the NPs seem to be encapsulated by a layer of liquid/gel. (f) CryoTEM image of the interface between two NPs. ~2 nm sized nanoparticulate texture was observed for the NPs. Liquid-like layers of pAH/CO_3^{2-} complex (see also Supplementary Fig. 14) could be found between the NPs, which show no detailed structure. The boundary of the NPs are highlighted by yellow dash lines. Scale bars: (a, d), 20 µm. (b, e), 200 nm. (c, f), 20 nm.

In order to check the adaptability of our observation in Fig. 2, the PILP processes induced by pAA or pAH were also examined. When 25 mg L^{-1} of pAA was used,[10] ACC thin films similar to those obtained in the pAsp system (Fig. 1) were formed on the glass slide (Supplementary Fig. 13a). ~50 nm sized NPs were found on the surface of the thin film by SEM (Supplementary Fig. 13b). CryoTEM shows the NPs possess ~2 nm sized nanoparticulate texture (Supplementary Fig. 13c). Similar structure was also found in a recent TEM study of a pAA/ACC hydrogel.[11] When 25 mg L^{-1} of pAH - a positively charge polymer - was used, again ACC thin films were formed (Supplementary Fig. 13d).[12] SEM image of the film showed ~200 nm sized NPs on its surface (Supplementary Fig. 13e), while cryoTEM showed the NPs seem to be encapsulated by a layer of liquid/gel (inset of Supplementary Fig. 13e). Zoom-in image of the interface between NPs showed the ~2 nm sized nanoparticulate texture of the NPs (Supplementary Fig. 13f), while no detailed structure was observed within the “liquid” layer between the NPs.

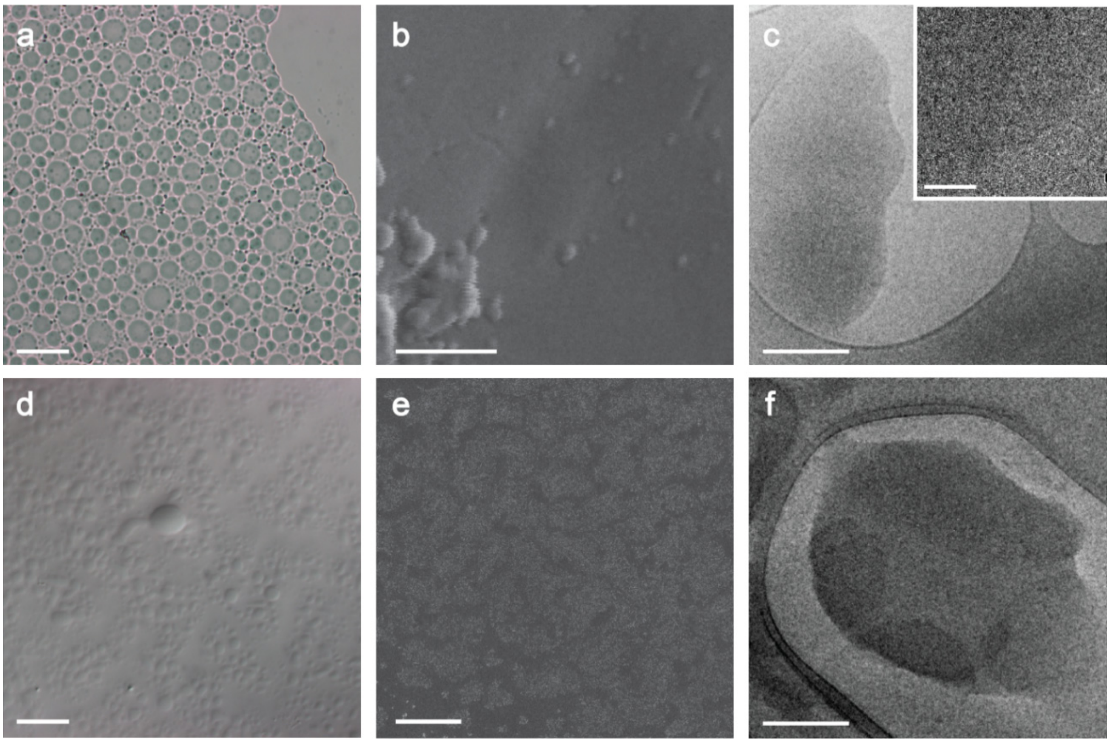

Supplementary Figure 14. Formation of pAH/CO_3^{2-} complex at higher concentration of pAH. (a-c) Product grown with 1000 mg/L pAH with 10 mM Ca^{2+}. (a) OM image showing the ACC thin film formed on the glass slide at 3 h, with micron sized spherical bumps on the film surface. (b) SEM image of the thin film, showing the film surface is relatively smooth. (c) CryoTEM image of the surface reaction solution at 3 h, showing liquid droplet-like objects. Inset is a zoom-image of the droplet, showing no detailed structure. (d-f) Solution containing 1000 mg L^{-1} of pAH and no Ca^{2+} after 3 h of $(NH_4)_2CO_3$ diffusion. (d) DIC-OM image of the micron sized spherical droplets formed on the glass slide at 3 h. (e) SEM image of the dried droplets. (f) CryoTEM image of the surface reaction solution at 3 h, showing liquid droplet-like objects similar to (c). Scale bars: (a, d, e), 20 µm. (b, c, f), 200 nm. Inset of (c), 20 nm.

When 1000 mg L^{-1} of pAH was used,[12] ACC thin films with micron-sized spherical bumps on the film surface were formed (Supplementary Fig. 14a). SEM showed the film was relatively smooth (Supplementary Fig. 14b) with only a few of nanoparticles. CryoTEM showed liquid-droplet-like objects similar to the study of Cantaert et al (Supplementary Fig. 14c).[12] These objects were continuous and did not show any nanosized detailed structure (inset of Supplementary Fig. 14c). Phase separation of pAH with presence of CO_3^{2-} and a range of different anions at weak alkaline conditions was previously reported,[12, 13] which is due to the formation and coexistence of R–NH_3^+ and R–$NHCO_2^-$ groups. To examine if the liquid-like object we observed is mainly due to the phase separation of pAH with CO_3^{2-}, a solution containing only 1000 mg L^{-1} of pAH with no Ca^{2+} was put into the $(NH_4)_2CO_3$ diffusion growth chamber for 3 h. Micron-sized spherical droplets were formed on the glass slide (Supplementary Fig. 14d), which were still visible after drying using SEM (Supplementary Fig. 14e). CryoTEM of the solution (Supplementary Fig. 14f) showed liquid droplet-like objects quite similar to those shown in Supplementary Fig. 14c. The results suggest that the liquid droplet-like objects we observed in the pAH system are mainly pAH/CO_3^{2-} coarcervate droplets. When high concentrations of pAH are used, high amounts of pAH/CO_3^{2-} coarcervate droplets are formed (Supplementary Fig. 14c and f) which cover on the products. The PILP NPs with the ~2 nm sized nanoparticulate texture were thus hidden during the cryoTEM observations (Supplementary Fig. 14c).[12] With lower pAH concentration, the PILP NPs can be observed (Supplementary Fig. 13e, f), however still to some extent covered by a thin layer of pAH/CO_3^{2-} complex.

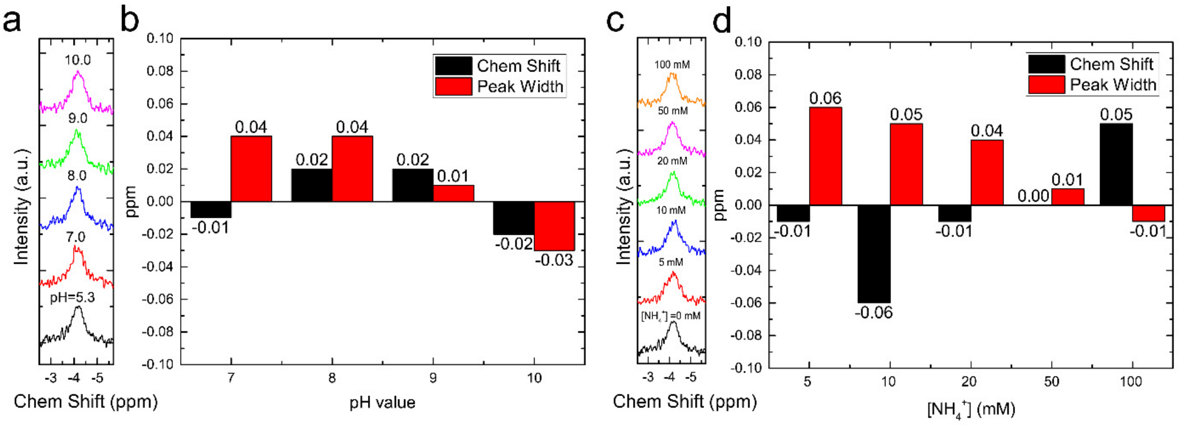

Supplementary Figure 15. ^{31}P liquid state NMR measurement of the solution containing 10 mM $CaCl_2$ and 2.5 g L^{-1} of ds-DNA measured with different pH values and [NH_4^+]. (a) Spectra of the solutions with pH=5.3, 7.0, 8.0, 9.0 and 10.0, respectively. The pH value was adjusted by 50 mM NaOH. (b) Changes of chemical shift and peak width of the ^{31}P signals upon the increase of pH value (compared with the original solution, pH=5.3). (c) Spectra of the solutions with [NH_4^+]=0, 5, 10, 20, 50 and 100 mM, respectively. The [NH_4^+] was introduced by mixing with NH_4Cl solutions. (d) Changes of chemical shift and peak width of the ^{31}P signals upon the increase of [NH_4^+] (compared with the original solution, [NH_4^+]=0 mM).

We have investigated the effect of pH value change to our ^{31}P liquid NMR measurements. Only a slight fluctuation of the chemical shift (~0.02 ppm) and peak width (~0.04 ppm) was observed when the pH value of our reaction solution (with 10 mM $CaCl_2$ and 2.5 g L^{-1} of ds-DNA) was raised from 5.3 to 10.0 by 50 mM NaOH, as shown in Supplementary Fig. 15a. This very small fluctuation may in fact be due to the intrinsic experimental variability of the measurements. So the pH will not have a significant effect on the chemical shift of ^{31}P signal during the reaction.

Nevertheless, we should consider that the generation of NH_4^+ ions due to the in-diffusion of NH_3 may influence the conformation of ds-DNA by changing the ionic strength. We investigated this by adding 5 to 100 mM of NH_4Cl into our reaction solution (Supplementary Fig. 15b). The addition of NH_4Cl indeed first shifts the ^{31}P signal to high field reaching a shift of $\Delta\delta$=-0.06 ppm at [NH_4^+] = 10 mM. When the concentration was further increased to 100 mM - which is close to the actual NH_4^+ concentration in the reaction solution,[14] the signal shifted back to 0.05 ppm down field of the initial solution.

We therefore attribute the small down field chemical shift observed after 200 min of the reaction (Fig. 4a and b) of 0.07 ppm (compared to the neat ds-DNA solution) mainly to the increase of the ionic strength and the influence of NH_4^+ on the conformation of ds-DNA.

Beyond this small shift, our results show a significant high field shift ($\Delta\delta$=-0.14 ppm) and broadening (0.27 ppm) of the ^{31}P signal of ds-DNA upon mixing with $CaCl_2$, and then a continuous down field shift (0.21 ppm) and sharpening (0.27 ppm) of the signal during the diffusion experiment. This trend is unlikely due only to the in-diffusion of NH_3, and should still be mainly attributed to the release of Ca^{2+} from binding with ds-DNA due to $CaCO_3$ formation.

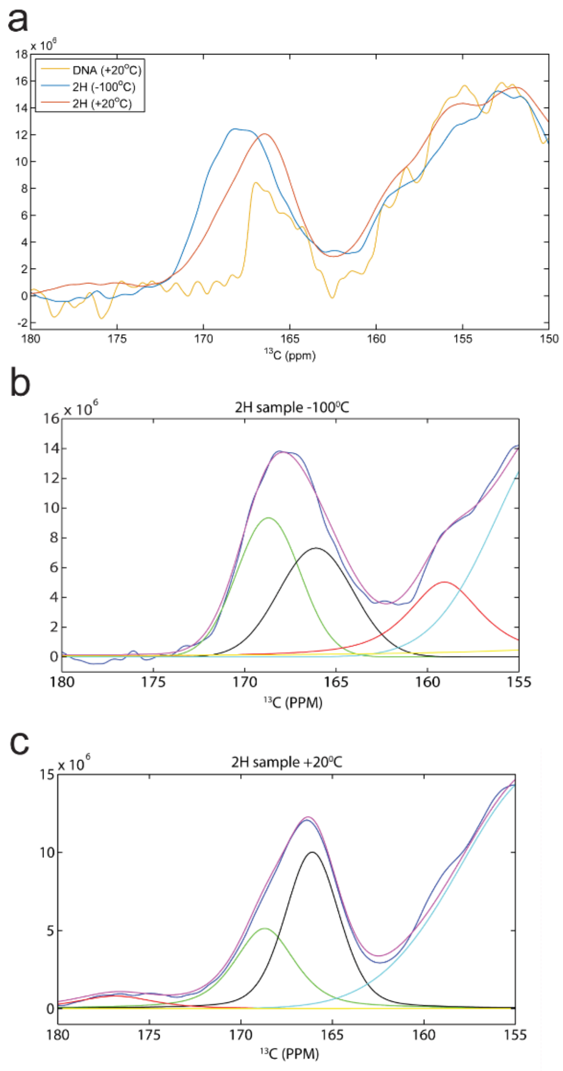

Supplementary Figure 16. (a) ^{13}C CP-MAS SS-NMR spectra of ds-DNA measured at 20 °C, and of the 2 hour sample measured at -100 and 20 °C, respectively. (b and c) The deconvoluted ^{13}C CP-MAS SS NMR spectra of the 2 hour sample measured at -100 and 20 °C, respectively

The ^{13}C CP-MAS SS-NMR spectrum of ds-DNA shows one peak at ~166 ppm within the range of 160-175 ppm, corresponding to the carbonyl groups of the base pairs in ds-DNA (Supplementary Fig. 16a).[15] The 2 hour sample showed a shoulder at the low field side of this peak, corresponding to the carbonate of ACC.[16] The signal of 2 hour sample between 160 and 175 ppm could be deconvoluted into two peaks at 168.7 ppm (peak 1, green) and 166.1 ppm (peak 2, black), corresponding to the carbonate of ACC and carbonyl groups of ds-DNA, respectively (Supplementary Fig. 16 b and c). The peak 1: peak 2 ratio was 1.10 at -100 °C and 0.62 at 20 °C, respectively. The different CP efficiency of ds-DNA and ACC indicates that local dynamics exist between the two components even in the solid-state, which influence the CP transfer for the ACC. At lower temperature, this dynamics is slowed down.

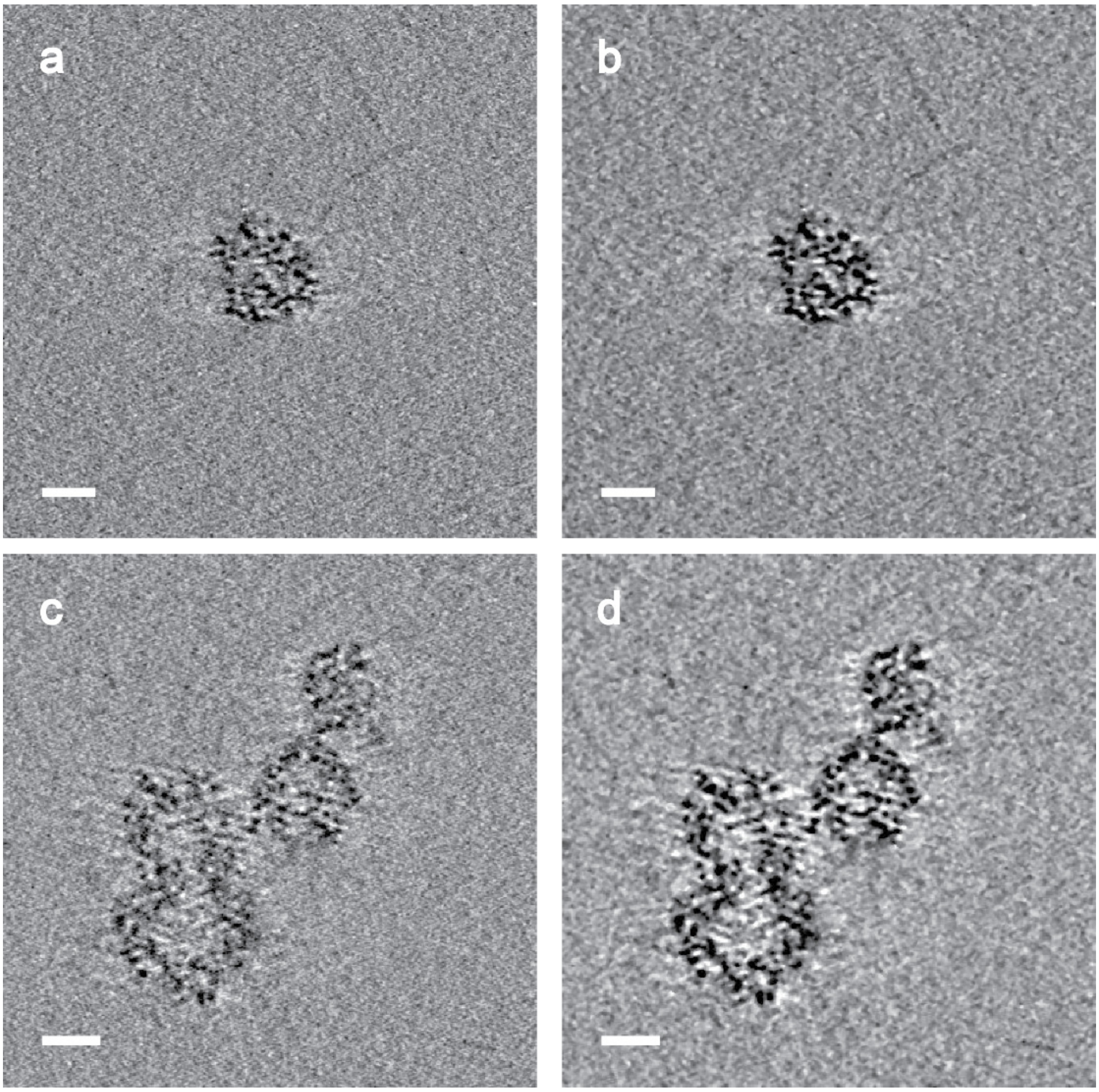

Supplementary Figure 17. Comparison between original and median filtered (filter size of 3x3x3 pixels) tomography cross section slices (shown in Fig. 2h and k) of ACC/ds-DNA NPs. (a) Original slice of NP (30 min) at Z=+4.6 nm. (b) Filtered slice of NP (30 min) at Z=+4.6 nm. (c) Original slice of hollow NPs (60 min) at Z=0 nm. (d) Filtered slice of hollow NPs (60 min) at Z=0 nm. Scale bars: 20 nm.

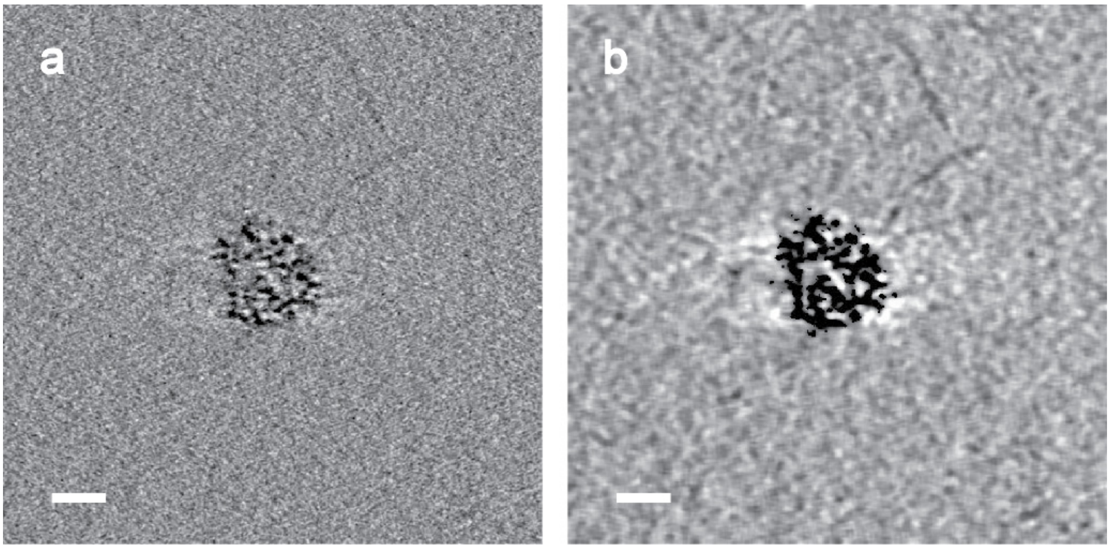

Supplementary Figure 18. Comparison between original and median filtered (mineralized area: filter size 3x3x 3 pixels, remaining area: filter size 7x7x7 pixels) and segmented tomography cross section slices of the ACC/ds-DNA NP (30min). (a) Original slice at Z=+4.6 nm. (b) Filtered and segmented slice at Z=+4.6 nm. Scale bars: 20 nm.

Supplementary Tables

	No additive	ds-DNA	pAsp
200 nm pore	2,33±1,16 µm	3,28±1,49 µm	5,66±2,11 µm
50 nm pore	1,27±0,74 µm	2,27±1,43 µm	2,77±0,47 µm

Supplementary Table 1. The lengths of $CaCO_3$ nanorods grown into the nanopores of track etched membrane. The nanorods were grown with no additive, 2.5 g L^{-1} of ds-DNA and 25 mg L^{-1} of pAsp, respectively. The data were obtained by measuring 20 nanorods for each sample and averaging. The ± values correspond to the standard deviations. The results show that ds-DNA and pAsp both promoted the growth, while pAsp was more effective than ds-DNA. Note that the amount of nanorods formed without additive is very small (<10% of those formed with presence of ds-DNA or pAsp).

	Ca (mg of sample g^{-1})	Ca (mg of sample g^{-1})	P (mg of sample g^{-1})	P (mg of sample g^{-1})	Mg (mg of sample g^{-1})	Mg (mg of sample g^{-1})	Na (mg of sample g^{-1})
Absorbance Wavelength	396.85 nm	317.933 nm	177.495 nm	178.287 nm	279.553 nm	280.270 nm	279.553 nm
**ds-DNA**	---	---	71.52±1.05	71.51±0.95	15.25±0.45	15.20±0.41	27.34±6.73
	Average value: ---		Average value: 71.52		Average value: 15.23		
**ds-DNA/Ca^{2+}**	40.45±0.21	40.15±0.25	61.62±2.92	61.13±2.55	0.93±0.04	0.92±0.04	---
	Average value: 40.30		Average value: 61.38		Average value: 0.93		
**2 h $CaCO_3$**	259.9±2.5	262.8±2.7	18.42±2.13	18.72±1.95	5.16±0.03	5.16±0.03	---
	Average value: 261.4		Average value: 18.57		Average value: 5.16		
**3 h $CaCO_3$**	349.1±7.8	351.7±7.3	4.57±0.28	4.46±0.28	4.84±0.01	4.85±0.01	---
	Average value: 350.4		Average value: 4.52		Average value: 4.85		
**5 h $CaCO_3$**	372.4±6.9	376.7±7.8	3.78±0.27	3.76±0.25	5.53±0.12	5.53±0.11	---
	Average value: 374.6		Average value: 3.77		Average value: 5.53		

Supplementary Table 2: The mass fractions measured by ICP-OES. Mass fractions of Ca, P, Mg and Na were measured in the ds-DNA, the ds-DNA/Ca^{2+} complex and $CaCO_3$ products grown with presence of ds-DNA and slow stirring (100 rpm) measured by ICP-OES. The data were obtained by averaging 10 measurements for each sample, while the ± values correspond to the standard deviations.

	Ca	P	Mg[a]	N[b]	Na	Cl
ds-DNA (EDS)	---	1	0.24	11.29	0.65	---
ds-DNA (ICP-OES)	---	1	0.272	N/A	0.521	N/A
ds-DNA/Ca^{2+} (EDS)	1	2.28	---	22.31	---	0.14
ds-DNA/Ca^{2+} (ICP-OES)	1	1.966	0.038	N/A	---	N/A
2 h $CaCO_3$ (EDS)	1	0.16	---	3.00	---	---
2 h $CaCO_3$ (ICP-OES)	1	0.092	0.032	N/A	---	N/A
3 h $CaCO_3$ (EDS)	1	0.03	---	---	---	---
3 h $CaCO_3$ (ICP-OES)	1	0.017	0.023	N/A	---	N/A
5 h $CaCO_3$ (EDS)	1	0.03	0.03	---	---	---
5 h $CaCO_3$ (ICP-OES)	1	0.013	0.024	N/A		N/A

a:The ds-DNA powder contained small amount Mg^{2+} and Na^{+}. Scarce Mg^{2+} was also detected in the $CaCO_3$ samples (5 h), due to the replacement of Mg^{2+} to Ca^{2+} in $CaCO_3$.[17]

b: The expected N/P ratio in ds-DNA IS ~3.75:1. The strong signal of N should be due to the relatively low accuracy of EDS when measuring light elements.[18]

Supplementary Table 3. The atom ratios measured by EDS and ICP-OES. Different elements in the ds-DNA, the ds-DNA/Ca^{2+} complex and $CaCO_3$ products grown with presence of ds-DNA and slow stirring (100 rpm) were measured by EDS and ICP-OES, respectively. In the Ca containing samples the ratio of Ca was set as 1, while in ds-DNA the ratio of P was set as 1 for the sake of comparison.

Pixel intensity threshold	-184	-284	-384	-484	-584
**Filtered (whole NP volume=394165 pix)**					
Volume of low pixel intensity area (pix)	236893	221915	201928	182498	158848
Proportion (%)	60.1	56.3	51.4	46.3	40.3
**Non-filtered (whole NP volume=352764 pix)**					
Volume of low pixel intensity area (pix)	212359	198118	183616	169347	154861
Proportion (%)	60.2	56.2	52.1	48.0	43.9

Supplementary Table 4. The volume proportion of the mineralized part of PILP. The volume proportion of the low pixel intensity (mineralized) part of the ACC/ds-DNA NP shown in Fig. 2g-j and Supplementary Movie 2 is measured. The values derived using different pixel intensity thresholds with or without filtering are compared in the table.

Ca^{2+} Concentration (mM)	Zeta Potential (mV), pH=5.3	Zeta Potential (mV), pH=10.0
0	-50.2±4.34	-53.1±6.01
1	-30.1±3.73	-33.9±3.99
2	-22.5±3.46	-26.2±3.68
5	-14.3±3.48	-20.3±4.26
10	-12.9±3.56	-15.9±5.56

Supplementary Table 5. Zeta potential of ds-DNA solutions containing Ca^{2+}. Zeta potentials were measured for 2.5 g L^{-1} ds-DNA solutions mixed with different concentrations of Ca^{2+}. The measurements were performed at pH=5.3 and 10.0, respectively. The zeta potential of pure ds-DNA solution was highly negative due to the negatively charged phosphate groups. The zeta potential significantly increased with increased Ca^{2+} concentration, indicating the binding between Ca^{2+} and ds-DNA. The zeta potentials measured at pH=10.0 are slightly lower than those measured at pH=5.3 due to the deprotonation at higher pH. The data were obtained by averaging 50 measurements of each sample, and the error bars correspond to the standard deviations.

ds-DNA Concentration (mg L^{-1})	Phosphate Group Concentration (mM)	Free [Ca^{2+}] (mM), pH=5.3	Bound [Ca^{2+}] (mM), pH=5.3	Free [Ca^{2+}] (mM), pH=10.0	Bound [Ca^{2+}] (mM), pH=10.0
0	0	10	0	10	0
25	0.07	9.65	0.35	9.67	0.33
66	0.18	9.57	0.43	9.52	0.48
250	0.67	9.26	0.74	9.31	0.69
666	1.77	8.85	1.15	8.97	1.03
2500	6.67	7.25	2.75	7.44	2.56

Supplementary Table 6. Free [Ca^{2+}] measurements of 10 mM $CaCl_2$ solution containing different concentrations of ds-DNA. The phosphate group concentration was estimated from the molecular weight (~225000) and number the number of base pairs (~300) of the ds-DNA. The measurements were performed at pH=5.3 and 10.0, respectively. It shows that the free Ca^{2+} in the solution decreased with increasing ds-DNA concentration. With 2.5 g L^{-1} of ds-DNA, only 7.25 mM of free Ca^{2+} was detected in the solution with pH=5.3, indicating 2.75 mM of Ca^{2+} was bound to ds-DNA. The free [Ca^{2+}] detected at pH=10.0 only deviate slightly with the values detected at pH=5.3, suggesting the binding between Ca^{2+} and ds-DNA is relatively strong and thus not significantly affected by the protonation/deprotonation of the phosphate groups.

Supplementary References

1. Addadi L, Moradian J, Shay E, Maroudas N, Weiner S. A chemical model for the cooperation of sulfates and carboxylates in calcite crystal nucleation: relevance to biomineralization. *Proc Nat Acad Sci* **84**, 2732-2736 (1987).

2. Addadi L, Raz S, Weiner S. Taking advantage of disorder: amorphous calcium carbonate and its roles in biomineralization. *Adv Mater* **15**, 959-970 (2003).

3. Kontoyannis CG, Vagenas NV. Calcium carbonate phase analysis using XRD and FT-Raman spectroscopy. *Analyst* **125**, 251-255 (2000).

4. Gauldie R, Sharma S, Volk E. Micro-Raman spectral study of vaterite and aragonite otoliths of the coho salmon, Oncorhynchus kisutch. *Compar Biochem Physiol A* **118**, 753-757 (1997).

5. Gower LB, Odom DJ. Deposition of calcium carbonate films by a polymer-induced liquid-precursor (PILP) process. *J Cryst Growth* **210**, 719-734 (2000).

6. Hermans TM*, et al.* Self-assembly of soft nanoparticles with tunable patchiness. *Nat Nanotechnol* **4**, 721 (2009).

7. van de Put MW*, et al.* Graphene oxide single sheets as substrates for high resolution cryoTEM. *Soft Matter* **11**, 1265-1270 (2015).

8. Dubochet J*, et al.* Cryo-electron microscopy of vitrified specimens. *Q Rev Biophys* **21**, 129-228 (1988).

9. Zhong C, Chu CC. Acid polysaccharide-induced amorphous calcium carbonate (ACC) films: colloidal nanoparticle self-organization process. *Langmuir* **25**, 3045-3049 (2009).

10. Hosoda N, Kato T. Thin-film formation of calcium carbonate crystals: effects of functional groups of matrix polymers. *Chem Mater* **13**, 688-693 (2001).

11. Sun S, Mao LB, Lei Z, Yu SH, Cölfen H. Hydrogels from amorphous calcium carbonate and polyacrylic acid: bio - inspired materials for "mineral plastics". *Angew Chem Int Ed* **55**, 11765-11769 (2016).

12. Cantaert B, Kim YY, Ludwig H, Nudelman F, Sommerdijk NA, Meldrum FC. Think positive: Phase separation enables a positively charged additive to induce dramatic changes in calcium carbonate morphology. *Adv Funct Mater* **22**, 907-915 (2012).

13. Brunner E, Lutz K, Sumper M. Biomimetic synthesis of silica nanospheres depends on the aggregation and phase separation of polyamines in aqueous solution. *Phys Chem Chem Phys* **6**, 854-857 (2004).

14. Ihli J, Bots P, Kulak A, Benning LG, Meldrum FC. Elucidating mechanisms of diffusion‐based calcium carbonate synthesis leads to controlled mesocrystal formation. *Adv Funct Mater* **23**, 1965-1973 (2013).

15. LaPlante SR*, et al.* Carbon-13 NMR of the bases of three DNA oligonucleotide duplexes: assignment methods and structural features. *Biochemistry* **27**, 7902-7909 (1988).

16. Nebel H, Neumann M, Mayer C, Epple M. On the structure of amorphous calcium carbonate ・ A detailed study by solid-state NMR spectroscopy. *Inorg Chem* **47**, 7874-7879 (2008).

17. Loste E, Wilson RM, Seshadri R, Meldrum FC. The role of magnesium in stabilising amorphous calcium carbonate and controlling calcite morphologies. *J Cryst Growth* **254**, 206-218 (2003).

18. Goldstein JI, Newbury DE, Michael JR, Ritchie NW, Scott JHJ, Joy DC. *Scanning electron microscopy and X-ray microanalysis*. Springer (2017).
