# Supplementary material for: Microscopic structure of the polymer-induced liquid precursor for calcium carbonate
Source: Nat Commun. 2018 Jul 3;9:2582. doi: 10.1038/s41467-018-05006-w (PMC6030133; doi:10.1038/s41467-018-05006-w)
Supplement: Supplementary file 3 — Description of Additional Supplementary Files [file 41467_2018_5006_MOESM3_ESM.pdf]

Description of Additional Supplementary Files

File Name: Supplementary Movie 1
Description: *In-situ* differential interference contrast optical microscopy (DICOM) visualization of the PILP induced ACC thin film formation process (with 25 mg L^{-1} of pAsp). The movie starts at 130 min and ends at 335 min after the reaction starts. The play back speed is 560 times of actual speed.

File Name: Supplementary Movie 2
Description: Cryogenic electron tomography of a PILP nanoparticle grown with 2.5 g L^{-1} of ds-DNA (30 min), showing its bicontinuous structure.

File Name: Supplementary Movie 3
Description: Cryogenic electron tomography of 4 attached PILP nanoparticles grown with 2.5 g L^{-1} of ds-DNA (60 min), indicating their hollow structure.
